# The Uptake and Use of Telemonitoring in Chronic Care Between 2014 and 2019: Nationwide Survey Among Patients and Health Care Professionals in the Netherlands

**DOI:** 10.2196/24908

**Published:** 2021-05-03

**Authors:** Martine W J Huygens, Helene R Voogdt-Pruis, Myrah Wouters, Maaike M Meurs, Britt van Lettow, Conchita Kleijweg, Roland D Friele

**Affiliations:** 1 NIVEL Netherlands Institute for Health Services Research Utrecht Netherlands; 2 Department of Global Health Julius Center for Health Sciences and Primary Care University Medical Center Utrecht Utrecht Netherlands; 3 National Institute for Public Health and the Environment Bilthoven Netherlands; 4 Nictiz The Hague Netherlands

**Keywords:** eHealth, telemonitoring, self-management, telemedicine, telehealth

## Abstract

**Background:**

Telemonitoring could offer solutions to the mounting challenges for health care and could improve patient self-management. Studies have addressed the benefits and challenges of telemonitoring for certain patient groups.

**Objective:**

This paper will examine the nationwide uptake of telemonitoring in chronic care in the Netherlands from 2014 to 2019 by means of an annual representative survey among patients and health care professionals.

**Methods:**

Between 2014 and 2019, approximately 2900 patients with chronic diseases, 700 nurses, and 500 general practitioners (GPs) and medical specialists received a questionnaire. About 30 questions addressed topics about the use of eHealth and experiences with it, including data about telemonitoring.

**Results:**

Between 2014 and 2019, the use of telemonitoring remained stable for all groups except medical specialists. In medical specialist departments, the use of telemonitoring increased from 11.2% (18/161) in 2014 to 19.6% (36/184) in 2019 (*χ*^2^_4_=12.3; *P*=.02). In 2019, telemonitoring was used by 5.8% (28/485) of people with chronic disease. This was 18.2% (41/225) in GP organizations and 40.4% (44/109), 38.0% (78/205), and 8.9% (29/325) in the organizations of nurses working in primary, secondary, and elderly care, respectively. Up to 10% of the targeted patient group such as diabetics were regarded by health care professionals as suitable for using telemonitoring. The main benefits mentioned by the patients were “comfort” (421/1043, 40.4%) and “living at home for longer/more comfortably” (334/1047, 31.9%). Health care professionals added “improvement of self-management” (63/176, 35.8% to 57/71, 80.3%), “better understanding of the patient’s condition” (47/176, 26.7% to 42/71, 59.2%), “reduction of workload” (53/134, 39.6% of nurses in elderly care), “better tailoring of care plan to the patient’s situation” (95/225, 42.2% of GPs), and “saves time for patients/caregivers” (61/176, 34.7% of medical specialists). Disadvantages mentioned by professionals were that “it takes time to monitor data” (13/130, 10% to 108/225, 48.0%), “it takes time to follow up alerts” (15/130, 11.5% to 117/225, 52.0%), and “it is difficult to estimate which patients can work with telemonitoring” (22/113, 19.5% to 94/225, 41.8%).

**Conclusions:**

The uptake of telemonitoring in Dutch chronic care remained stable during 2014-2019 but increased among medical specialists. According to both patients and professionals, telemonitoring improves the quality of life and quality of care. Skills for suitably including eligible patients and for allocating the tasks of data monitoring and follow-up care within the team would help to further increase the use of telemonitoring.

## Introduction

### Added Value of Telemonitoring

Telemonitoring could broaden access to health care and offer solutions to the mounting challenges for the health care system such as an ageing population, which is creating a demand for long-term care, rising expectations from patients who are better informed about health issues, and the pressure on national health care budgets due to these demands [1-4]. Telemonitoring uses technology such as videoconferencing, email, remote electronic monitoring equipment, social network apps, and internet portals to allow monitoring and self-monitoring of health data by patients and health-related education and long-distance interventions by health care professionals (HCPs) [5-7]. Several studies have addressed the benefits of telemonitoring, for example, better access to health care and the cost-effective delivery of health care. Telemonitoring could reduce face-to-face consultations and clinic visits. In addition, telemonitoring improves the quality of care and clinical outcomes through continuous and reliable monitoring of data, immediate assessment, triage, and interventions. Telemonitoring could also improve patient empowerment, self-management, and compliance [4,8-16].

### Implementation of Telemonitoring

Implementation and actual use of telemonitoring in daily practice require well-thought-out action plans, for example, for selecting appropriate interventions or for tailoring the design to the needs of the user group. In addition, telemonitoring should be seamlessly integrated into the health care processes and should avoid disrupting the HCPs’ existing workflow. It was also recommended that telemonitoring should be part of “blended care” as both patients and HCPs prefer face-to-face encounters [17,18]. Implementation programs for telemonitoring should tackle barriers to actual use. Some studies found barriers that are related to “users” of telemonitoring such as lack of digital skills, resistance to change, and lack of direct personal benefit. Mentioned examples of barriers that were related to the context were lack of privacy (or fear of it), security, patient safety, a properly working internet connection, proper technological infrastructure, regulations, funding, and task allocation [4,19].

### Telemonitoring in the Netherlands

In the Netherlands, an enabling factor related to the context of telemonitoring is the availability of 4G mobile networks and high-speed broadband Internet access, even in rural areas [20]. Currently, 90% of Dutch people use the internet daily. In particular, over the last 5 years, older people, the less well-educated, those born outside the country, and low-income households have caught up [21]. In terms of policy, several national documents, studies, and guidelines for eHealth have been developed during the last 10 years addressing privacy and patient safety in the use of eHealth [22,23]. In 2012, the Dutch National Implementation Agenda for eHealth was launched, followed by the eHealth Governance Covenant 2014 - 2019 [24] and a framework on the use of eHealth by HCPs [23]. As regards funding, telemonitoring is covered by the Dutch health insurance system; in particular, the funding of follow-up consultations improved in 2019. Still, HCPs do not have the funds to upscale telemonitoring [25].

### Annual Nationwide Representative Study Among Health Care Professionals

Since 2013, the nationwide uptake of eHealth in general by patients and HCPs has been investigated annually and reported in what is known as the “eHealth-monitor.” This investigation was commissioned by the Dutch Ministry of Health, Welfare and Sport. The aim of the annual eHealth-monitor was to investigate the implementation of eHealth and to boost its implementation in subsequent health care policy. Every year, about 30 questions addressed topics on the use of eHealth and experiences with it, including telemonitoring. The findings from the perspectives of nurses, general practitioners (GPs), medical specialists, and patients with a chronic disease increased the understanding of the implementation of telemonitoring and the uptake of telemonitoring in daily practice. To our knowledge, our study is the first scientific paper on the nationwide uptake of telemonitoring for all patient groups in chronic care over a long period of time. We analyzed data on the actual use of telemonitoring by patients and HCPs in daily chronic care. In addition, opinions on and experiences with telemonitoring were analyzed.

## Methods

### Study Design

Since 2013, data for the eHealth-monitor has been collected annually using various nationwide panels. Written and online questionnaires on eHealth were sent to GPs, medical specialists, nurses (practice nurses and practice assistants working in elderly care, GP care, and hospital care), and people with chronic diseases. All participants were approached in March. Nonresponders initially received 1 written or 2 online reminders. For this study, data from respondents on questions about telemonitoring between 2014 and 2019 were used.

### Study Population

People with chronic diseases may be included in the representative National Panel of people with Chronic illness or Disability (NPCD) [26]. Inclusion criteria for the NPCD are age 15 or older, diagnosed with a somatic chronic disease, aware of the diagnosis, having a life expectancy of more than 6 months, mentally capable of participating, and not permanently institutionalized. Every year, 500 new panel members are selected to replace panel members who have withdrawn or who have participated for 4 years. The questions on telemonitoring were posed in 2015, 2017, and 2019.

Nurses are participants in a representative nursing staff panel. The nursing staff panel consists of a nationwide group of nursing staff members (nurses, caregivers, and practice assistants) in various health care settings who deliver direct patient care. The recruitment of members of the nursing staff panel takes place through a random sample of two pension funds. Together, these pension funds register all employees in the Dutch health care sector. Nursing staff were asked to participate in health care research for various purposes. People who agreed and who delivered direct nursing care to patients could join the nursing staff panel. The questions on telemonitoring were asked annually, from 2014 until 2019. For this study, the data of nurses working in primary care, secondary care, and elderly care were used. For 2014 until 2016, the data of nurses working in the curative sectors (practice nurses and nurses working in hospital care) were taken together. From 2017 onwards, these sectors were split into two samples.

General practitioners and medical specialists are participants in a representative doctor’s panel. Included are all registered GPs and medical specialists of the Royal Dutch Medical Association. Inclusion criteria for participating in the eHealth-monitor were practicing in the past year and being involved in the diagnosis or treatment of patients. From these doctors, certain specializations were excluded: public and occupational health, forensic medicine, addiction medicine, and psychiatry. The questions on telemonitoring were asked annually, from 2014 until 2019.

### Questionnaires

All participants were asked about their use of telemonitoring and experience with its advantages and disadvantages in the previous 12 months. In addition, HCPs were asked for which portion of patients telemonitoring was used and for which patient groups relevant (Textbox 1).

**Questionnaires.**

**Telemonitoring**: Remote monitoring of a patient, in which they measure their own health values (for example, blood pressure, blood sugar level) using a meter, sensor, or other device in the home situation and in which they could also respond to some questions. The HCP receives these data digitally.

**People with chronic diseases who measure health values themselves**

- Which of the following statements apply to you? (multiple answers possible)
- I electronically submit my self-measured health values to my health care provider (eg, by email or automatically via computer or mobile app) (2015, 2017, 2019)
- My health care provider can see my health data on a website or in a mobile app (2015, 2017, 2019)
- My health care provider looks at my self-measured health data before or during a consultation and discusses it with me (2015, 2017, 2019)
- My health care provider keeps an eye on my self-measured health data remotely and contacts me if anything is wrong (2015, 2017, 2019)
- Can you say how desirable or necessary telemonitoring is for you? (2017)
- Could you please answer the following statements? (2019) I notice or think that telemonitoring...
  ...makes it easier for me to live at home longer and/or more easily
  ...takes a lot of effort for me
  ...improves my care
  ...makes me very tense

**Nurses**

- Has telemonitoring been used in your organization in the past year? (2014-2019)
- If so, what proportion of your clients/patients use telemonitoring (estimated)? (2016, 2017, 2019)
- If so, what proportion of your clients do you think telemonitoring makes sense for (estimated)? (2019)
- What advantages and disadvantages do you experience with telemonitoring or expect from it? (multiple answers possible) (2019)

**General practitioners**

- Could you state whether telemonitoring is applied to the following patient groups (in your practice)? If telemonitoring is not applied, could you please state whether there are plans to start within a year and whether you would like to do so? (2014-2017)
- In your practice, some of your patients with diabetes are currently being monitored by telemonitoring. Could you please estimate the proportion of your diabetes patients for whom you think telemonitoring is sensible? What proportion of your diabetes patients do use it? (2015-2017)
- Is telemonitoring of patients relevant to you or your practice, and is it used within your practice for some or all of the patients? (2019)
- Could you estimate the size of the group of patients for whom you think telemonitoring is sensible and for whom you or other HCPs actually use it in your practice? (2019)
- What advantages and disadvantages do you experience with telemonitoring or expect from it? (multiple answers possible) (2019)

**Medical specialists**

- Is telemonitoring of patients relevant to your medical specialty, and is it applied by your department for some or all patients? (2014-2017, 2019)
- For which group of your patients is telemonitoring relevant? (2015-2017)
- Could you estimate the size of the group of patients for whom you think telemonitoring is sensible and for whom you or other HCPs actually use it at your department? (2015-2017, 2019)
- What advantages and disadvantages do you experience with telemonitoring or expect from it? (multiple answers possible) (2019)

### Data Analysis

Descriptive analyses were conducted to study the use and experiences of telemonitoring. Data from the questionnaires among people with a chronic disease and nursing staff were analyzed using Stata, version 15.0 (StataCorp). The results from the questionnaires among GPs and medical specialists were analyzed using SPSS, version 25.0 (IBM Corp).

For questions asked to people with chronic diseases, the descriptive analyses were weighted for age and gender in such a way that it resembled the distribution of age and gender within the Dutch population from age 18 years, based on data from Statistics Netherlands. We applied a weighting factor ranging from 0.65 to 2.28. The samples for nurses and GPs are fairly representative of the Dutch population of nurses and GPs regarding gender, but the response is not representative for age: GPs younger than 35 years and GPs and nurses 50 years and older responded more often. Nevertheless, we did not use a weight factor to correct for this because applying the weight factor did not affect the results. For questions asked to medical specialists, the descriptive analyses were weighted for type of specialty. We applied a weighting factor ranging from 0.5 to 1.7.

## Results

### Participants

Over the years, data were used from 485-633 people with a chronic disease, 322-607 nurses working in elderly care, 220-367 nurses working in the curative disciplines (primary and secondary care), 225-396 GPs, and 184-386 medical specialists who answered questions about telemonitoring (Table 1). The mean age of people with chronic disease was 64.5 to 66.4 years. The mean age of nurses varied from 46.7 to 52.2 years. The mean ages of GPs and medical specialists ranged from 50.0 to 52.6 years and 49.4 to 53.9 years, respectively. Approximately half of the people with chronic disease and the doctors were men; among nurses, only 3.5% (21/607) to 16.1% (25/155) were men.

**Table 1:** Study population per year.^a^

Characteristic		2014	2015	2016	2017	2018	2019
**People with chronic disease**							
	Responses, n	—^b^	1448	—	1357	—	1292
	Telemonitoring responses, n^c^	—	604	—	633	—	485
	Male, n (%)	—	315 (52.2)	—	304 (48.0)	—	234 (48.2)
	Age (years), mean (SD)	—	64.9 (12.7)	—	66.4 (12.1)	—	64.5 (11.9)
	Low education, n (%)	—	133 (22.0)	—	156 (24.6)	—	95 (19.6)
**Nurses in elderly care**							
	Telemonitoring responses, n	408	607	433	341	322	325
	Male, n (%)	18 (4.4)	21 (3.5)	20 (4.6)	15 (4.4)	29 (9.0)	19 (5.8)
	Age (years), mean (SD)	50.8 (8.9)	48.9 (10.8)	49.7 (10.3)	52.2 (9.5)	50.1 (10.8)	50.9 (11.1)
**Nurses in curative sectors**							
	Telemonitoring responses, n	262	316	220	—	—	—
	Male, n (%)	26 (9.9)	36 (11.4)	25 (11.4)	—	—	—
	Age (years), mean (SD)	48.8 (9.6)	46.7 (11.4)	48.4 (10.7)	—	—	—
**Practice nurses**							
	Telemonitoring responses, n	—	—	—	212	124	109
	Male, n (%)	—	—	—	12 (5.7)	8 (6.5)	8 (7.3)
	Age (years), mean (SD)	—	—	—	50.8 (8.6)	50.4 (9.0)	52.0 (9.1)
**Nurses in hospital care**							
	Telemonitoring responses, n	—	—	—	155	211	205
	Male, n (%)	—	—	—	25 (16.1)	29 (13.7)	27 (13.2)
	Age (years), mean (SD)	—	—	—	49.4 (11.0)	50.0 (11.2)	48.2 (12.4)
**General practitioners**							
	Telemonitoring responses, n	—	396	316	290	—	225
	Male, n (%)	—	194 (49.0)	162 (51.3)	133 (45.9)	—	106 (47.1)
	Age (years), mean (SD)	—	50.0 (10.2)	51.1 (9.9)	51.5 (9.7)	—	52.6 (9.1)
**Medical specialists**							
	Telemonitoring responses, n^d^	—	386	274	253	—	184
	Male, n (%)	—	203 (52.6)	151 (55.1)	153 (60.5)	—	118 (64.1)
	Age (years), mean (SD)	—	49.8 (11.3)	49.4 (11.1)	51.9 (11.3)	—	53.9 (9.7)

^a^Among general practitioners and medical specialists, the questions in 2015, 2016, and 2017 were different from those in 2019.

^b^Not available.

^c^Question was only asked to persons who measured health outcomes by themselves.

^d^Weighted by type of specialty.

### Actual Use of Telemonitoring

In 2019, 5.8% (28/485) of people with a chronic disease stated that their HCP monitors the health values remotely and contacts them if anything looks wrong (Table 2). In 2015, this figure was 3.3% (20/604). Most of them had been diagnosed with cardiovascular disease or diabetes. In 2019, 38.0% (78/205) of nurses working in secondary care and 40.4% (44/109) in primary care stated that their organization uses telemonitoring. In 2014, this percentage was 34.0% (89/262) for nurses working in the curative disciplines (both hospital and general practice nurses). Among nurses working in elderly care this was 8.9% (29/325) in 2019 and 11.3% (46/408) in 2014. In addition, 18.2% (41/225)of GPs and 19.6% (36/184)of medical specialists stated that telemonitoring was used in their organizations in 2019. These percentages were 17.0% and 11.2%, respectively, in 2014. Among medical specialists, this number has grown significantly over the years (*χ*^2^_4_=12.3, *P*=.02). Up to 10% of the targeted patient group, including people with diabetes, is reckoned to be suitable for using telemonitoring.

**Table 2:** Proportions of patients and health care professionals (HCPs) using telemonitoring, from 2014 to 2019.

Population using telemonitoring			2014		2015		2016		2017		2018		2019		
Patients with a chronic disease, n (%)^a^			—^b^		20 (3.3)		—		37 (5.8)		—		28 (5.8)		
**Elderly care**															
	Nurses, n (%)^c^	46 (11.3)		70 (11.5)		—		—		—		29 (8.9)			
	Patients using telemonitoring, n (mode %)^d^	—		—		27 (1-10)		25 (1-10)		—		17 (1-10)			
**Primary/secondary care**															
	Nurses, n (%)	89 (34.0)		126 (39.9)		84 (38.2)		—		—		—			
	Patients using telemonitoring, n (mode %)	—		—		18 (1-10)		—		—		—			
**Primary care**															
	Nurses, n (%)	—		—		—		85 (40.1)		58 (46.8)		44 (40.4)			
	Patients using telemonitoring, n (mode %)	—		—		—		61 (1-10)		—		25 (1-10)			
**Secondary care**															
	Nurses, n (%)	—		—		—		50 (32.3)		80 (37.9 )		78 (38.0)			
	Patients using telemonitoring, n (mode %)	—		—		—		14 (1-10)		—		22 (1-10)			
**General practitioners, n (%)^c^**			29 (17.0)		49 (12.4)		41 (13.0)		26 (9.0)		—		41 (18.2)		
	Group using telemonitoring, n (mode group)	25 (Diabetes)		43 (Diabetes)		37 (Diabetes)		21 (Diabetes)		—		—			
	Relevant for diabetes, n (mode %)^d^	—		26 (1-10)		20 (1-10)		10 (1-10)		—		—			
**Medical specialists, n (%)^c^**			18 (11.2)		41 (10.6)		26 (9.5)		29 (11.5)		—		36 (19.6)		
	Relevant for patients, n (mode %)^d^	—		24 (1-10)		19 (1-10)		—		—		—			
	Relevant for patient group, n (mode group)^e^	—		13 (Diabetes)		9 (Diabetes)		11 (Diabetes)		—		—			

^a^The proportion of responding patients stating they are monitored on self-reported health measures by HCP (remotely).

^b^Not available.

^c^The proportion of responding HCPs stating that telemonitoring is relevant or is used at their department, practice, or organization. Difference between years (*χ*^2^_4_=12.3; *P*=.02).

^d^Proportion of patients using telemonitoring according to the HCPs; possible answers: none, up to 10%, up to 20%, up to 50%, up to 100%, I don’t know.

^e^The most often mentioned were these groups: diabetes, heart failure, chronic obstructive pulmonary disease, and asthma.

### Advantages and Disadvantages of Telemonitoring

Of patients with a chronic disease, 40.4% (421/1043) agreed or totally agreed with the statement “telemonitoring improves my comfort” (353/1043, 33.8% answered “don’t know”). In addition, 31.9% (334/1047) agreed or totally agreed with “telemonitoring lets me stay at home longer and/or live more comfortably” (405/1050, 38.7% answered “don’t know”) (Table 3).

On the other hand, 7.7% agreed or totally agreed with “telemonitoring takes me a lot of effort” (36.4% answered “don’t know”). Among HCPs, the most widely experienced or expected advantage of telemonitoring mentioned was “telemonitoring improves patients’ self-management” (57/71, 80.3% of nurses working in primary care; 71/124, 57.3% of nurses working in secondary care; 84/134, 62.7% of nurses working in elderly care; 135/225, 60% of GPs; 63/176, 35.8% of medical specialists) (Table 4). In addition, 59.2% (42/71) of nurses working in primary care and 46.8% (58/124) of nurses working in secondary care stated that their experience or expectation of telemonitoring was that they would get a better understanding of the health condition of the patient. Of nurses working in elderly care, 39.6% (53/134) experienced or expected telemonitoring to reduce the workload. Moreover, 42.2% (95/225) of GPs said that telemonitoring let them tailor the care plan to the situation of their patients better. Of medical specialists, 34.7% (61/176) expected or had observed that telemonitoring gave them spare time for patients and caregivers/relatives.

**Table 3:** Opinions of telemonitoring from patients with chronic diseases (n=1023-1050), 2019.

I notice or think that telemonitoring:	Agreement with statement, n (%)					
	Totally disagree	Disagree	Neither agree nor disagree	Agree	Totally agree	I don’t know
Lets me live at home longer and/or more easily (n=1047)	47 (4.5)	62 (5.9)	199 (19.0)	235 (22.4)	99 (9.5)	405 (38.7)
Improves my comfort (n=1043)	42 (4.0)	50 (4.8)	177 (17.0)	331 (31.7)	90 (8.6)	353 (33.8)
Takes me a lot of effort (n=1023)	83 (8.1)	249 (24.3)	246 (24.0)	61 (6.0)	12 (1.2)	372 (36.4)
Improves my care (n=1035)	41 (4.0)	66 (6.4)	234 (22.6)	241 (23.3)	82 (7.9)	371 (35.8)
Makes me very tense (n=1027)	104 (10.1)	220 (21.4)	238 (23.2)	77 (7.5)	17 (1.7)	371 (36.1)

**Table 4:** Experienced or expected advantages and disadvantages of telemonitoring assessed by health care providers, 2019.

Characteristic			Nurses, primary care		Nurses, secondary care		Nurses, elderly care		General practitioners		Medical specialists
Advantages respondents, n			71		124		134		225		176
**Advantages, n (%)^a^**											
	It reduces the workload	10 (14.1)		23 (18.5)		53 (39.6)		16 (7.1)		14 (8.0)	
	It reduces the workload of my assistants	—^b^		—		—		56 (24.9)		18 (10.2)	
	It saves time for patients and or caregivers/relatives	32 (45.1)		58 (46.8)		45 (33.6)		86 (38.2)		61 (34.7)	
	It improves the quality of care in my organization	34 (47.9)		50 (40.3)		38 (28.4)		77 (34.2)		44 (25.0)	
	It improves the self-management of the patient	57 (80.3)		71 (57.3)		84 (62.7)		135 (60.0)		63 (35.8)	
	I have a better understanding of my clients’ health condition	42 (59.2)		58 (46.8)		43 (32.1)		77 (34.2)		47 (26.7)	
	It lets me tailor the care plan better to my patients’ situation	38 (53.5)		47 (37.9)		32 (23.9)		95 (42.2)		45 (25.6)	
	It lets patients ask for help in time	20 (28.2)		52 (41.9)		38 (28.4)		67 (29.8)		32 (18.2)	
	Other advantages	—		7 (5.6)		3 (2.2)		9 (4.0)		5 (2.8)	
	I do not expect or experience any advantages	—		9 (7.3)		23 (17.2)		32 (14.2)		48 (27.3)	
Disadvantages respondents, n			68		113		130		225		181
**Disadvantages, n (%)^a^**											
	It takes me a lot of time to monitor/check health values	32 (47.1)		21 (18.6)		13 (10.0)		108 (48.0)		56 (30.9)	
	It takes me a lot of time to follow up notifications	29 (42.6)		19 (16.8)		15 (11.5)		117 (52.0)		51 (28.2)	
	It takes a lot of time for my assistants	—		—		—		99 (44.0)		45 (24.9)	
	It ensures that patients and/or relatives contact me more often	20 (29.4)		22 (19.5)		21 (16.2)		83 (36.9)		40 (22.1)	
	It worries patients and/or relatives	10 (14.7)		20 (17.7)		23 (17.7)		81 (36.0)		33 (18.2)	
	I find it difficult to work with it	—		—		—		16 (7.1)		7 (3.9)	
	The system provides unreliable data	—		—		—		52 (23.1)		33 (18.2)	
	The application is not secure	—		—		—		20 (8.9)		7 (3.9)	
	I find it difficult to estimate which patients can work with it	15 (22.1)		22 (19.5)		37 (28.5)		94 (41.8)		52 (28.7)	
	Other disadvantages	8 (11.8)		7 (6.2)		8 (6.2)		18 (8.0)		20 (11.0)	
	I do not expect or experience any disadvantages	13 (19.1)		49 (43.4)		56 (43.1)		13 (5.8)		33 (18.2)	

^a^Multiple answers possible. ^b^Not available.

A total of 43.4% (49/113) of nurses working in secondary care and 43.1% (56/130) of nurses working in elderly care did not expect or experience any disadvantages of telemonitoring. In contrast, 47.1% (32/68) of nurses working in primary care expected or noted that telemonitoring takes a lot of time to monitor and check health values. Of GPs and medical specialists, 48.0% (108/225) and 30.9% (56/181), respectively, expected or experienced this. In addition, 42.6% (29/68) of nurses working in primary care, 52.0% (117/225) of GPs, and 28.2% (51/181) of medical specialists expected or experienced that using telemonitoring would take up a lot of time following up on alerts. Moreover, 19.5% (22/113) of nurses working in secondary care, 28.5% (37/130) of the nurses working in elderly care, and 28.7% (52/181) of medical specialists expected or experienced difficulties in estimating which patients could handle telemonitoring.

## Discussion

### Principal Findings

Our study adds new insights to current scientific studies of telemonitoring, as it investigated the actual nationwide uptake of telemonitoring for all patient groups in chronic care over a long period of time (before the COVID-19 pandemic). Findings from the perspectives of nurses, GPs, medical specialists, and patients with chronic diseases can assist the implementation of telemonitoring and the uptake of telemonitoring in daily practice. The current COVID-19 pandemic has called for rapid implementation of telemonitoring for acute and subacute diseases in 2020. Future editions of the Dutch eHealth-monitor might present the impact of COVID-19 on a rising uptake of telemonitoring [27] and on new opinions and experiences with telemonitoring. Strengths of our study include the large sample size, the external validity and reliability of the data, and the representativeness of the various groups of participants (people with chronic diseases, nurses, GPs, and medical specialists). Nevertheless, there are also some limitations. Due to the specific factors in the Netherlands that boosted the implementation of telemonitoring, our study results can only partly be extrapolated to other countries. In addition, the use of telemonitoring was only investigated by asking potential users in health care; we did not investigate data from other resources such as health care insurers or telehealth companies. As well, our quantitative approach is best suited to answering “what,” “when,” and “who” questions and less well-suited to “how” and “why” questions. The opinions and experiences with telemonitoring that we investigated therefore do not fully explain the factors concerning the implementation and uptake of telemonitoring. Even so, our results concerning the experiences of HCPs are underlined by qualitative studies [28-30]. Other benefits and barriers found are “an increased feeling of safety” and “insufficient familiarity with the technology” [28-30]. In addition, HCPs need to add telemonitoring to the health care process, with a precise description of the target group, task allocation for data monitoring, and support for patients from within the team [31,32].

### Conclusion

The uptake of telemonitoring in Dutch chronic care remained stable during 2014-2019 but increased among medical specialists. According to both patients and professionals, telemonitoring improves the quality of life and quality of care. Skills for appropriately including eligible patients and allocating the tasks of data monitoring and follow-up care within the team would help to further increase the use of telemonitoring.

## Abbreviations

GP: general practitioner
HCP: health care professional
NPCD: National Panel of people with Chronic illness or Disability
